# Grey matter abnormalities in methcathinone abusers with a Parkinsonian syndrome

**DOI:** 10.1002/brb3.539

**Published:** 2016-08-18

**Authors:** Julius Juurmaa, Ricarda A. L. Menke, Pierre Vila, Andreas Müürsepp, Tiiu Tomberg, Pilvi Ilves, Mait Nigul, Heidi Johansen‐Berg, Michael Donaghy, Charlotte J. Stagg, Ainārs Stepens, Pille Taba

**Affiliations:** ^1^ Department of Neurology and NeurosurgeryUniversity of TartuTartuEstonia; ^2^ Nuffield Department of Clinical Neurosciences (NDCN)Centre for Functional Magnetic Resonance Imaging of the Brain (FMRIB)University of OxfordOxfordUK; ^3^ Department of PsychiatryUniversity of OxfordOxfordUK; ^4^ Radiology ClinicTartu University HospitalTartuEstonia; ^5^ NDCNUniversity of OxfordOxfordUK; ^6^ Laboratory for Research in RehabilitationRiga Stradins UniversityRigaLatvia

**Keywords:** ephedrone, manganese toxicity, methcathinone, MRI, Parkinsonism

## Abstract

**Background:**

A permanent Parkinsonian syndrome occurs in intravenous abusers of the designer psychostimulant methcathinone (ephedrone). It is attributed to deposition of contaminant manganese, as reflected by characteristic globus pallidus hyperintensity on T1‐weighted MRI.

**Methods:**

We have investigated brain structure and function in methcathinone abusers (*n* = 12) compared to matched control subjects (*n* = 12) using T1‐weighted structural and resting‐state functional MRI.

**Results:**

Segmentation analysis revealed significant (*p* < .05) subcortical grey matter atrophy in methcathinone abusers within putamen and thalamus bilaterally, and the left caudate nucleus. The volume of the caudate nuclei correlated inversely with duration of methcathinone abuse. Voxel‐based morphometry showed patients to have significant grey matter loss (*p* < .05) bilaterally in the putamina and caudate nucleus. Surface‐based analysis demonstrated nine clusters of cerebral cortical thinning in methcathinone abusers, with relative sparing of prefrontal, parieto‐occipital, and temporal regions. Resting‐state functional MRI analysis showed increased functional connectivity within the motor network of patients (*p* < .05), particularly within the right primary motor cortex.

**Conclusion:**

Taken together, these results suggest that the manganese exposure associated with prolonged methcathinone abuse results in widespread structural and functional changes affecting both subcortical and cortical grey matter and their connections. Underlying the distinctive movement disorder caused by methcathinone abuse, there is a more widespread pattern of brain involvement than is evident from the hyperintensity restricted to the basal ganglia as shown by T1‐weighted structural MRI.

## 1 Introduction

Due to the ease of synthesis from readily available components methcathinone (ephedrone) still presents a continuing public health hazard in many Eastern European countries. Intravenous abuse of this designer psychostimulant results in a levodopa‐unresponsive Parkinsonian syndrome (Stepens et al., 2014). Home preparation of methcathinone by oxidation of the pseudoephedrine and ephedrine in common cold medications, in the presence of potassium permanganate, results in a high overload of manganese. The movement disorder closely resembles that of chronic manganism, involving combinations of hypokinesia, dysarthria, dystonia, and postural instability which manifest as facial impassivity, slowed movements, low volume speech, micrographia, lurching gait with absent arm swing, and falls (Stepens et al., 2008). This methcathinone/manganese‐associated movement disorder can be severely disabling, is permanent, and shows no improvement despite abstinence (Selikhova et al., 2008; Sikk et al., 2007; Stepens et al., 2008).

In active users of methcathinone, manganese blood levels are markedly elevated and associated with a distinctive T1‐weighted MRI signal hyperintensity in the basal ganglia (Selikhova et al., 2008; Sikk et al., 2007; Stepens et al., 2008), which is attributable to manganese deposition (Klos et al., 2006). Diffusion‐weighted MR tractography shows abusers to have diffuse white matter abnormalities throughout the brain, with severe focal damage in tracts underlying the right ventral premotor cortex and the medial prefrontal cortex. These findings point to a widespread neuropathology underlying this disorder of higher level motor programming (Stepens et al., 2010). So far no studies have investigated grey matter pathology either within the basal ganglia or elsewhere in the brain. This MRI study investigates subcortical and cortical grey matter integrity in intravenous methcathinone abusers.

## 2 Methods

### 2.1 Subject recruitment and clinical testing

Twelve intravenous drug abusers (mean age 32.4 years [range 22.4–44.6]; eight men) who had self‐reported using methcathinone for a mean period of 3.7 years (range 0.5–12), all showing the typical movement disorder, and 12 healthy age‐ and sex‐matched controls (mean age 31.7 years [range 23.6–39.0]; eight men) underwent MRI. Abstinent users were defined by self‐reported abstinence for ≥1 year, and by absence of globus pallidus hyperintensity on T1‐weighted MRI. Informed consent was obtained in accordance with the Declaration of Helsinki and ethical approval was granted by the Research Ethics Committee of the University of Tartu and the Ethical Committee of Riga Stradins University.

Disability was quantified using the Movement Disorder Society‐sponsored revision of the Unified Parkinson's Disease Rating Scale (MDS‐UPDRS; Goetz et al., 2008) which includes the Hoehn‐Yahr (Hoehn & Yahr, 1967) and Schwab‐England scales (Schwab & England, 1969). Quality of life was assessed using the Parkinson's Disease Quality of Life Questionnaire (PDQ‐39; Jenkinson, Fitzpatrick, Peto, Greenhall, & Hyman, 1997) and depressive symptoms were documented using the Beck's Depression Inventory (Beck, Ward, Mendelson, Mock, & Erbaugh, 1961). The Mini‐mental State Examination (MMSE) was used to examine cognitive state (Folstein, Folstein, & McHugh, 1975). Olfaction was tested using the Sniffin' Sticks (SS‐12) smell test (Hummel, Konnerth, Rosenheim, & Kobal, 2001). Higher scores indicate more severe symptomatology on all clinical tests, except the Schwab‐England scale and MMSE.

### 2.2 Image acquisition

All MRI data were acquired using a MR scanner 3.0T Achieva (Philips Medical Systems) at Tartu University Hospital. Whole‐brain T1‐weighted scans were acquired using a 3D Fast Field Echo sequence with 146 axial slices (TR = 12 ms, TE = 3.2 ms, 1.0 mm isotropic resolution).

Resting‐state fMRI data were acquired with dynamic T2*‐weighted gradient echo single shot EPI‐BOLD sequence (TR = 3,000 ms, TE = 35 ms, acquisition voxel size 3.0 mm isotropic). Sixty volumes were acquired with 47 axial slices.

### 2.3 Image analysis

All image analysis was performed using tools from the FMRIB Software Library version 4.1 (FSL; Smith et al., 2004; www.fmrib.ox.ac.uk/fsl; RRID:SCR_002823) and FreeSurfer version 5.1 (Dale, Fischl, & Sereno, 1999; surfer.nmr.mgh.harvard.edu; RRID:SCR_001847).

#### 2.3.1 Assessment of volume differences of subcortical grey matter structures

We segmented putamen, caudate, pallidum, and thalamus from each subject's T1‐weighted image using FMRIB's Integrated Registration and Segmentation Tool (Patenaude, Smith, Kennedy, & Jenkinson, 2011). Furthermore, for each subject, brain tissue volume, normalized for subject head size, was estimated with SIENAX (Smith et al., 2002), part of FSL. SIENAX starts by extracting brain and skull images from the single whole‐head input data. The brain image is then affine registered to MNI152 space (using the skull image to determine the registration scaling); this is primarily in order to obtain the volumetric scaling factor, to be used for normalization for head size. Tissue‐type segmentation with partial volume estimation is carried out in order to calculate total volume of brain tissue (including separate estimates of volumes of total grey matter, white matter, cortical grey matter, and ventricular CSF).

The results of each step of the image processing, most importantly the subcortical segmentation, were carefully examined to ensure accuracy of the results. As expected, the signal hyperintensities in the left and right pallidum prevented correct automated (as well as reliable manual) segmentation in the sub‐group of patients who were active users; the pallidum was therefore excluded from volumetric analysis.

Before conducting statistical analyses, the volumes of each subcortical region of interest were adjusted for inter individual head size differences via multiplication by the volumetric scaling factor derived from SIENAX. All statistical analyses were carried out using IBM SPSS Statistics (Version 20). Statistical comparisons were carried out separately for each hemisphere using independent samples t‐tests. Pearson correlation coefficients were calculated to investigate the relationship between adjusted subcortical volumes and clinical rating scale scores.

#### 2.3.2 Voxel‐based morphometry

T1‐weighted MPRAGE data were analyzed using a standard approach with FSL voxel‐based morphometry (VBM; part of FSL), a voxel‐based morphometry style analysis (Ashburner & Friston, 2000; Good et al., 2001). First, structural images were brain extracted (Smith, 2002). Next, tissue‐type segmentation was carried out using FAST4 (Zhang, Brady, & Smith, 2001). The resulting grey matter partial volume images were then aligned to MNI152 standard space using the affine registration tool FLIRT (Jenkinson, Bannister, Brady, & Smith, 2002; Jenkinson & Smith, 2001), followed by nonlinear registration using FNIRT (Anderson, Andersson, Jenkinson, & Smith, 2007). The resulting images were averaged to create a study‐specific template, to which the native grey matter images were then nonlinearly reregistered. We then multiplied the registered partial volume images of all subjects by the Jacobian of the warp field (“modulation”) to correct for local expansion or contraction. The modulated segmented images were smoothed with an isotropic Gaussian kernel with a sigma of 3 mm.

To investigate group differences, a voxel‐wise GLM was applied using permutation‐based nonparametric testing with correction for multiple comparisons (family‐wise error [FWE]; Smith & Nichols, 2008).

#### 2.3.3 Cerebral cortical thickness analysis

Cortical reconstruction and volumetric segmentation were performed via a semiautomated process using the FreeSurfer image analysis suite. This processing includes removal of nonbrain tissue using a hybrid watershed/surface deformation procedure (Segonne, Pacheco, & Fischl, 2007), automated transformation to Talairach space, segmentation of the subcortical white matter and deep grey matter volumetric structures (Fischl et al., 2002, 2004), intensity normalization (Sled, Zijdenbos, & Evans, 1998), tessellation of the grey matter/white matter boundary, automated topology correction (Fischl, Liu, & Dale, 2001; Segonne et al., 2007), and surface deformation following intensity gradients to demarcate the grey matter/white matter and grey matter/CSF borders at the location where the greatest shift in intensity defines the transition to the other tissue class (Dale et al., 1999; Fischl & Dale, 2000). For two subjects, manual adjustment of watershed parameters for the skull stripping procedure was required; four subjects needed additional control points for complete tracing of brain tissue; and for one subject, tracing of the pial surface had to be corrected. Finally, completed cortical models were resampled into a common space (Fischl & Dale, 2000). Computations were carried out in the High Performance Computing Center of the University of Tartu.

Anatomical landmarks encompassed by clusters with statistically significant changes were labeled according to a complete parcellation of the cortical surface that uses internationally accepted standard nomenclature and criteria (Destrieux, Fischl, Dale, & Halgren, 2010). Graphical reconstructions were created using PySurfer.

Cortical thickness measurements by FreeSurfer have been extensively validated against histological analysis (Rosas et al., 2002) and manual measurements (Kuperberg et al., 2003; Salat et al., 2004).

Cortical thickness was compared firstly between patients and controls and then between active and abstinent users using mri_glmfit, part of the FreeSurfer toolkit. Inference was performed using permutation testing and the maximum cluster size as the test statistic, allowing correction for multiple comparisons across surface vertices. A *z*‐threshold corresponding to *p* < .05 was chosen for both the cluster‐forming threshold and cluster‐wise significance threshold.

#### 2.3.4 Resting‐state functional MRI analysis

Resting‐state fMRI analysis was carried out using the Multivariate Exploratory Linear Optimised Decomposition into Independent Components tool (Beckmann, DeLuca, Devlin, & Smith, 2005), part of FSL. First, standard preprocessing steps were performed on each individual subject's fMRI images, consisting of motion correction, brain extraction, and spatial smoothing using a Gaussian kernel of full‐width at half‐maximum of 6 mm, and high‐pass temporal filtering equivalent to 150 s. Next, fMRI images were registered to the individual's structural images using boundary‐based linear registration and then to standard MNI space images using FNIRT (Anderson et al., 2007).

Preprocessed functional data containing 60 time points for each subject were temporally concatenated across subjects to create a single 4D dataset. Concatenated fMRI multiple datasets were decomposed using independent component analysis (ICA) to give 25 spatial components. Subsequently, components corresponding to the eight canonical resting‐state networks (RSN) were selected (Beckmann et al., 2005). A dual‐regression approach was used to identify, within each subject's fMRI dataset, subject‐specific temporal dynamics, and associated spatial maps. This involved spatial regression of group‐ICA spatial maps against individual fMRI datasets, resulting in matrices describing temporal dynamics for each component and subject, followed by temporal regression of time‐course matrices against fMRI data to estimate subject‐specific spatial maps. Finally, different component maps (corresponding to a specific RSN) in each subject were concatenated into single 4D files.

To investigate group differences, a voxel‐wise GLM was applied using permutation‐based nonparametric testing, with FWE correction for multiple comparisons (Smith & Nichols, 2008). We specifically studied the motor network, using the default‐mode network (DMN) as an anatomical control. The DMN is distinct from the motor network and there is no evidence of its involvement in this clinical syndrome.

In order to test for altered strength of functional coupling between the deep grey structures, functional connectivity was investigated based on regional correlations of the BOLD signal time courses. The representative time course of activity for each structure (pallidum, putamen, caudate, and thalamus in MNI space) was extracted. Functional connectivity was calculated using Pearson correlation coefficients between different structures, converted into *Z* statistics using Fisher's *r*‐to‐*Z* transformation, and these resulting statistics were used to test for group differences between patients and controls using a Repeated Measures ANOVA.

## 3 Results

### 3.1 Patient characteristics

Clinical rating scale results are summarized in Table 1. The patients' MMSE scores demonstrated normal cognitive function and SS‐12 scores indicated normal olfaction. MDS‐UPDRS III (motor examination) subscores show patients had a significant impairment of motor function.

**Table 1:** Patients' clinical rating scale results

Clinical test and component		Scale	Mean ± *SE*
MDS‐UPDRS			
I	Nonmotor aspects of daily living	0–52	11.8 ± 3.5
II	Motor aspects of daily living	0–52	15.5 ± 3.8
III	Motor examination	0–132	35.8 ± 9.5
Total		0–236	63.0 ± 11.9
Hoehn‐Yahr scale		0–5	3.5 ± 0.5
Schwab‐England scale		0–100	65.0 ± 9.5
MMSE		0–30	28.9 ± 0.8
PDQ‐39			
I	Mobility	0–100	49.6 ± 16.6
II	Activities of daily living	0–100	28.8 ± 10.8
III	Emotional well‐being	0–100	41.7 ± 11.9
IV	Stigma	0–100	46.4 ± 15.6
V	Social support	0–100	38.9 ± 12.3
VI	Cognitive impairment	0–100	25.0 ± 13.0
VII	Communication	0–100	51.4 ± 13.9
VIII	Bodily discomfort	0–100	42.4 ± 11.3
Total		0–100	40.5 ± 6.5
SS‐12		0–12	9.4 ± 0.7

### 3.2 Subcortical structure volume

Volume was significantly reduced in patients compared to controls for putamina and thalami bilaterally (right putamen: *p* = .036, left putamen: *p* = .019, right thalamus: *p* = .034, left thalamus: *p* = .015), and for the left caudate nucleus (*p* = .037).

**Figure 1:**
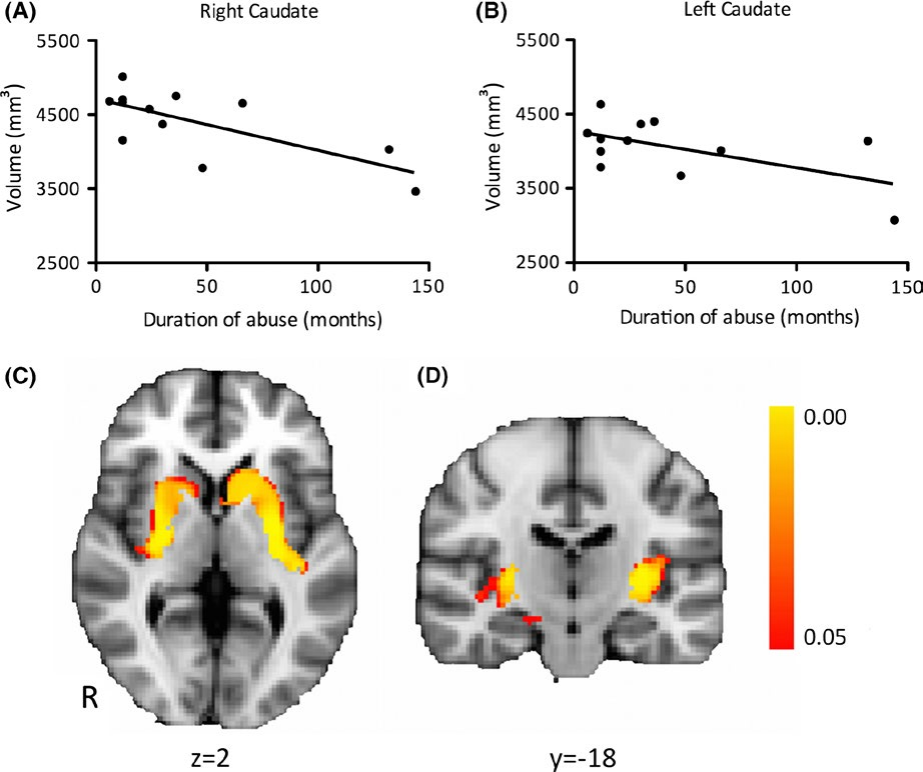
Negative correlation between duration of methcathinone abuse and caudate nucleus volume (A and B). Regions (caudate and putamen bilaterally) showing significant (*p*_corr_ < .05) grey matter atrophy in patients as compared with controls (C and D). *y*/*z* = MNI coordinates

The duration of methcathinone use correlated negatively with the volume of the right caudate nucleus (*r* = −.717, *p* = .009; Fig. 1A). Furthermore, there was a trend toward significant negative correlation between duration of methcathinone use and the volume of the left caudate nucleus (*r* = −.576, *p* = .05; Fig. 1B).

### 3.3 Voxel‐based morphometry

Whole‐brain VBM analysis (excluding the globus pallidus) revealed areas of significant grey matter loss in patients compared to controls in the left and right putamen and caudate, as well as in the deep left temporal lobe (Fig. 1C and D). No correlations with duration of methcathinone use were demonstrated.

### 3.4 Cerebral cortical thickness

**Figure 2:**
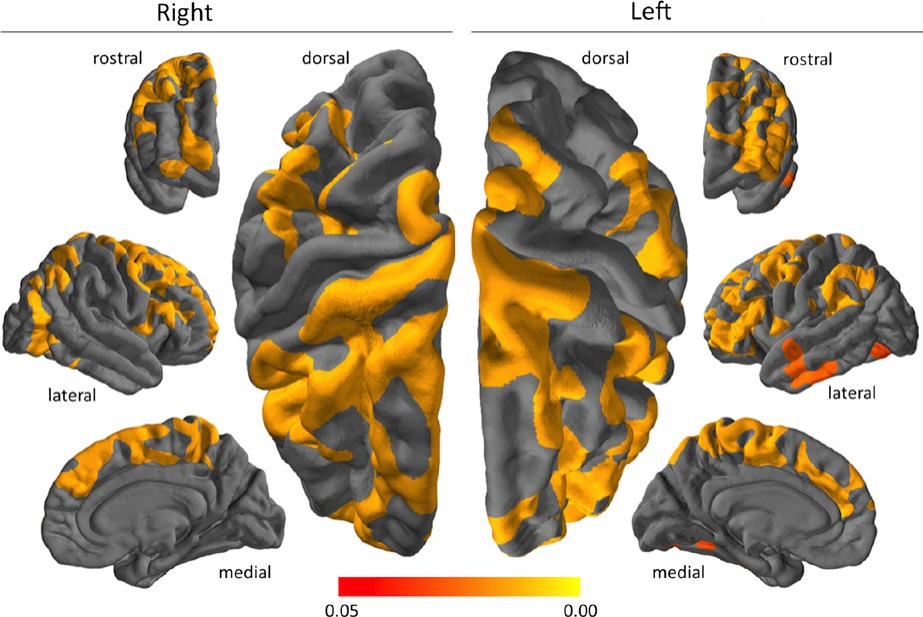
Clusters of significant cerebral cortical thinning in methcathinone abusers compared to controls. The color grading shows differing levels of statistical significance; highlighted areas all exceed the threshold of *p* < .05, corrected for multiple comparisons

When compared to controls, patients displayed cortical thinning in a total of nine clusters (Fig. 2, Table 2). Notably, given the preserved MMSE scores, large areas of prefrontal, parietal, and temporal cortex were relatively spared.

**Table 2:** Regions of reduced cortical thickness in patients compared with controls

	Area, mm^2^	Landmarks	Cluster‐wise *p*
L	5,038	Superior frontal gyrus and sulcus Precentral gyrus and sulcus Paracentral lobule and sulcus Superior parietal lobule	.0002
L	5,711	Superior frontal sulcus Middle frontal gyrus and sulcus Fronto‐marginal gyrus (of Wernicke) and sulcus Orbital gyrus and lateral orbital sulcus Inferior frontal gyrus and sulcus	.0002
L	3,199	Angular gyrus Supramarginal gyrus Posterior ramus of the lateral sulcus	.0002
L	1,298	Superior temporal sulcus Middle temporal gyrus Inferior temporal gyrus and sulcus	.0016
L	1,494	Precentral gyrus and sulcus Middle frontal gyrus	.0002
L	1,213	Lateral occipito‐temporal gyrus and sulcus Medial occipito‐temporal sulgus and lingual sulcus Inferior occipital gyrus and sulcus	.0030
L	1,409	Superior frontal gyrus Anterior and middle part of the cingulate gyrus and sulcus	.0022
R	11,357	Superior frontal gyrus and sulcus Middle frontal gyrus Inferior frontal gyrus and sulcus Fronto‐marginal gyrus (of Wernicke) and sulcus Precentral gyrus and sulcus Paracentral gyrus and sulcus Marginal branch of the cingulate sulcus Middle part of the cingulate gyrus and sulcus	.0002
R	4,985	Superior parietal lobule Angular gyrus Anterior occipital sulcus Inferior occipital gyrus and sulcus Inferior temporal gyrus and sulcus Lateral occipito‐temporal gyrus and sulcus	.0002

Comparison of active (*n* = 5) and abstinent (*n* = 7) methcathinone users revealed less cortical thinning in active users in two clusters, comprising the postcentral gyrus and sulcus, superior parietal lobule, and the intraparietal sulcus and transverse parietal sulci of the left hemisphere (*p* = .016), and the central sulcus, postcentral gyrus, and sulcus, and the supramarginal gyrus in the right hemisphere (*p* = .023). There was no significant difference in the duration of methcathinone usage between active and abstinent users.

### 3.5 Resting‐state functional connectivity

**Figure 3:**
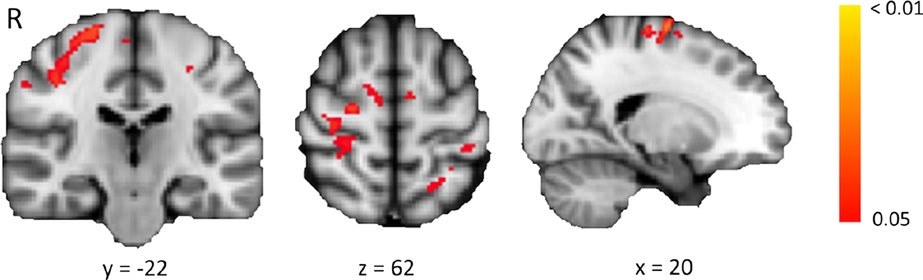
Group differences in the motor resting‐state networks between patients and controls. Red‐yellow regions indicate voxels showing a significant increase in functional connectivity in patients compared with controls. Color bar indicates *p*‐values, corrected for multiple comparisons. *x*/*y*/*z* = MNI coordinates

We investigated functional connectivity within the motor network while the patients were at rest. Voxel‐wise comparison revealed several regions with significantly increased coactivation with the motor RSNs in patients compared to controls (*p* < .05), mainly within the right primary motor cortex (Fig. 3). No differences were seen in the control DMN.

There were no significant differences between patients and controls in functional connectivity between the basal ganglia structures (RM‐ANOVA main effect of group [patients vs. controls; *F*(1, 22) = 0.337, *p* = .567]).

## 4 Discussion

This is the first comprehensive study examining structural integrity and functional connectivity of cortical and subcortical grey matter structures in intravenous methcathinone abusers. Subcortical structure volumetry and whole‐brain voxel‐based grey matter morphometry both showed significant striatal differences bilaterally between patients and controls. Furthermore, volumetric comparison revealed reduced thalamic volume in methcathinone abusers. In addition to these changes affecting subcortical structures, surface‐based analysis demonstrated widespread areas of cerebral cortical thinning in patients. Resting‐state functional MRI analysis revealed significantly increased functional connectivity at rest within the motor network in methcathinone abusers, particularly within the primary motor cortices bilaterally.

The observed pattern of subcortical grey matter loss is most likely to reflect the manganese toxicity associated with intravenous methcathinone abuse. Neuropathological changes in manganese toxicity are mainly limited to basal ganglia structures; neurodegeneration and reactive changes have been observed in the globus pallidus, striatum, subthalamic nucleus, and substantia nigra pars reticulata (Aschner, Erikson, Herrero Hernandez, & Tjalkens, 2009). Damage to these basal ganglia structures is in keeping with the patients' Parkinsonian clinical features. In particular, damage to the putamen, the most consistent site of grey matter loss in our study, is associated with motor symptomatology (Middleton & Strick, 2000). Unfortunately, the MRI T1‐weighted signal hyperintensity attributable to manganese deposition within the globi pallidi precluded reliable segmentation within the patient group, preventing assessment of morphometric group differences in this structure. Notably, our previous study of methcathinone abusers demonstrated increased mean diffusivity in the globus pallidus and a decrease in fractional anisotropy within globus pallidus–cortical connections (Stepens et al., 2010).

Functional coupling between brain regions, in part reflecting underlying anatomical connectivity, can be measured using resting‐state functional connectivity. The cause of the increased functional connectivity we demonstrated in the motor network remains unclear. This pattern of structural atrophy associated with increased functional connectivity has been described in a number of motor disorders, including Parkinson's disease (Poston & Eidelberg, 2012) and amyotrophic lateral sclerosis (Douaud, Filippini, Knight, Talbot, & Turner, 2011). This increase in functional connectivity in conjunction with structural atrophy may reflect either a compensatory adaptation response to injury, or a loss of inhibitory neuronal influences resulting from structural damage (Douaud et al., 2011).

Does methcathinone itself, in addition to the manganese toxicity, contribute to the observed brain changes, as well as the clinical symptoms, of these patients? Animal models have shown that methcathinone acts mainly on monoaminergic systems outside the extrapyramidal motor network (Gygi, Fleckenstein, Gibb, & Hanson, 1997; Gygi, Gibb, & Hanson, 1996). Human imaging studies showed normal dopamine levels and intact presynaptic nigrostriatal neurons in cases of methcathinone/manganese‐induced movement disorder (Sikk et al., 2010); suggesting manganese rather than methcathinone as the main causative agent of movement disorder in our patients. Methcathinone is an “amphetamine‐like” drug and methcathinone and amphetamine have similar chemical structures (Glennon, Yousif, Naiman, & Kalix, 1987). The most consistently observed change associated with chronic amphetamine abuse is reduced cerebral cortical grey matter density and volume, along with enlargement of the globus pallidus and putamen (Berman, O'Neill, Fears, Bartzokis, & London, 2008). It seems that the structural changes caused by chronic amphetamine abuse mainly affect the cortex, whereas manganese toxicity predominantly produces subcortical damage. It may be, therefore, that intravenous methcathinone abuse is a “double toxin” with manganese toxicity affecting the subcortical structures and the methcathinone component leading to the widespread changes in cerebral cortical thickness. It is worth noting, however, that a similar pattern of widespread cortical thinning has been seen in progressive supranuclear palsy (Worker et al., 2014), a disease that shares many clinical features with those seen in our cohort of methcathinone abusers. This finding would suggest that the cortical thinning observed in our patients may be a result of neurodegeneration secondary to the subcortical damage caused by manganese toxicity rather than the direct effect of methcathinone per se, although a histopathological study would be required to confirm or refute these hypotheses.

## 5 Conclusion

We have demonstrated widespread atrophy and increased functional connectivity within the brains of methcathinone abusers with a manganese‐induced movement disorder. These abnormalities occurred both within and outside the basal ganglia. The damage localized to subcortical structures within the motor loop of the basal ganglia is likely to be attributable to manganese toxicity and probably underlies the distinctive nature of this stereotyped motor syndrome. This notion is supported by the resting‐state fMRI analysis revealing increased functional connectivity within the motor network. Our identification of diffuse cerebral cortical thinning may reflect an additional toxic effect of methcathinone, by analogy with the effects of amphetamine, a similar stimulant. Overall our results illustrate that in intravenous methcathinone/manganese abusers there is a more widespread pattern of grey matter damage than demonstrable by conventional imaging techniques.

## Funding Information

National Research Program of Latvia (Grant/Award Number: 5.8.2) and Estonian Science Foundation (Grant/Award Number: GARLA0148P, GARNR9199).

## Conflict of Interest

Nothing to disclose.
